# Discordant results of Xpert MTB/Rif assay and BACTEC MGIT 960 liquid culture to detect *Mycobacterium tuberculosis* in community screening in Vietnam

**DOI:** 10.1186/s12879-022-07481-5

**Published:** 2022-05-31

**Authors:** Hai Viet Nguyen, Petra de Haas, Hoa Binh Nguyen, Nhung Viet Nguyen, Frank G. J. Cobelens, Veriko Mirtskhulava, Alyssa Finlay, Hung Van Nguyen, Pham T. T. Huyen, Edine W. Tiemersma

**Affiliations:** 1 National Tuberculosis Programme, 463 Hoang Hoa Tham, Ba Dinh District, Hanoi, Vietnam; 2 KNCV Tuberculosis Foundation, The Hague, the Netherlands; 3 Department of Global Health and Amsterdam Institute of Global Health and Development, Amsterdam University Medical Centres location University of Amsterdam, Amsterdam, the Netherlands; 4 David Tvildiani Medical University, Tbilisi, Georgia; 5 Centers for Disease Control - Vietnam Office, Hanoi, Vietnam

**Keywords:** Tuberculosis, TB, Xpert, MGIT culture

## Abstract

**Background:**

Xpert MTB/Rif, a molecular test to detect tuberculosis (TB), has been proven to have high sensitivity and specificity when compared with liquid culture in clinical settings. However, little is known about its performance in community TB screening.

**Methods:**

In Vietnam, a national TB prevalence survey was conducted in 2017. Survey participants who screened positive by chest X-ray, cough symptoms and/or recent history of tuberculosis were requested to provide at least two sputum samples that were tested for *Mycobacterium tuberculosis* by Xpert MTB/Rif G4 (Xpert) and BACTEC MGIT960 culture (MGIT).

**Results:**

There were 4,649 eligible participants provided both samples for testing. Among them, 236 (5.1%) participants tested positive for TB by Xpert, 244 (5.3%) tested positive by MGIT and 317 tested positive by at least one test; 163 (51.4%) had discordant test results. Of the positive Xpert, 162 (68.6%) showed a low or very low bacterial load. In multivariate logistic regression comparing discordant with Xpert-MGIT concordant positive results, discordant Xpert-positive results occurred more often among participants who had low sputum bacterial load, male sex, a history of TB treatment, or night sweats. The associated factors were male sex, abnormal chest X-ray and having night sweats when the logistic model was against those with both Xpert and MGIT negative.

**Conclusions:**

We found high rates of discordance in the performance of Xpert and MGIT for community-based TB case finding. In situations where the majority of TB cases are expected to have a low bacterial load, multiple diagnostic tests and/or multiple samples are required to reach sufficient sensitivity.

**Supplementary Information:**

The online version contains supplementary material available at 10.1186/s12879-022-07481-5.

## Background

Despite global efforts, a large gap remains for the detection of tuberculosis (TB). In 2019, there were an estimated ten million new TB patients worldwide, while only six million were diagnosed and notified [1]. This diagnostic gap is caused by poor clinical awareness, stigma in community settings, and the lack of rapid, sensitive and accessible diagnostics [2]. Sputum smear microscopy has been the mainstay of TB detection on a national scale in lower-middle-income countries, as it is rapid, inexpensive and widely applicable [3]. However, its sensitivity for TB diagnosis ranges from 34 to 80%, leaving vast numbers of TB cases undetected [4, 5]. The BD BACTEC Mycobacteria Growth Indicator Tube 960 (MGIT) system, a liquid medium-based culture, is regarded as the reference standard to diagnose TB, due to its high accuracy and exceptional sensitivity and specificity [6]. However, liquid culture is often only available in specialized facilities, has long times to detection and high rates of contamination, inhibiting large-scale use for prompt treatment of TB patients [7, 8].

The World Health Organization (WHO) has recommended using rapid molecular assays such as Cepheid Xpert MTB/Rif (Xpert) for TB diagnosis in specified patients groups [9]. Xpert is an automated within-cartridge PCR that produces results within two hours. Numerous studies have shown that Xpert performs well compared with liquid culture for symptomatic patients in clinical settings, with its sensitivity to detect *Mycobacterium tuberculosis* (MTB) ranging from 82 to 95% [10–13].

Because of its short turnaround time and limited labour requirements compared to culture, Xpert is potentially also an ideal TB diagnostic to use in community TB screening. Scale-up of active case finding approaches such as community screening is considered important to further reduce the global TB burden [14, 15]. However, little is known about the performance of Xpert in community screening. Compared to clinical settings, community screening is characterised by a lower prevalence of TB, more asymptomatic TB cases and on average lower bacterial load in the sample. The latter may limit the sensitivity of Xpert to diagnose TB [16, 17]. In addition, while culture detects only live bacilli, Xpert identifies MTB DNA, including non-viable bacilli that may remain detectable after treatment of previous TB disease. These differences raise the question of the usefulness of Xpert in community TB screening.

In 2017, the Vietnam National TB Programme (NTP) conducted a nationwide TB prevalence survey to assess the TB burden in the country, using Xpert and MGIT to detect TB [18]. We aimed to assess the utility of Xpert in community TB screening by comparing the results of Xpert and MGIT testing done in this survey and assessing the factors associated with discordances between results of Xpert and MGIT.

## Methods

### Study design and population

The second national TB prevalence survey in Vietnam was conducted from October 2017 to February 2018, using multistage cluster sampling to select and screen 71,748 participants from 82 communities across the country [18]. Participants were screened for TB by oral interview and digital chest X-ray, those with a screened-positive result were further interviewed to explore their TB-related symptoms. Also, they were requested to provide sputum samples for MTB testing using Xpert and MGIT. A TB presumptive participant, or “screened-positive” and thus eligible for sputum examination, was defined as a participant who had cough for 2 weeks or more; a self-reported history of TB treatment in the 2 years preceding the survey; or chest X-ray abnormalities consistent with TB. The present analysis was restricted to those who screened positive for TB and had valid Xpert and MGIT results. Survey participants currently on TB treatment or had a TB treatment history within the preceding 2 years were excluded from the analysis, as TB treatment produces non-viable MTB bacilli making discordance more likely to occur [11]. Participants whose test results were inadequate, i.e. missing Xpert or MGIT result, or MGIT result was contaminated or non-tuberculous mycobacteria, were also excluded to ensure the consistency of the analysis.

### Sputum collection and testing procedures

Participants screened positive were requested to provide at least two sputum samples, an on-the-spot sample (S) and an early morning sample (M). The M sample was collected right after waking up in the morning of the next day in Falcon tubes. The S sample was examined using Xpert (G4 cartridge) at the survey field site or transported in iceboxes to a nearby district laboratory on the same day. For the initial S sample (S1), if the Xpert result was positive for MTB, or the test was unsuccessful and there was not enough S1 sample left for re-testing, the participant was asked to provide an additional S sample (S2) to run a repeat Xpert test when handing over the morning sample on the next day. The M samples were kept in portable refrigerators at 2–8 °C and delivered to the National Reference Laboratory or one of three regional reference laboratories for culture.

In the survey site Xpert laboratories, when the S samples arrived, after checking for an adequate amount of sputum, laboratory technicians had added Xpert sample reagent (SR) into the S sample tubes with the SR:sputum ratio of 2:1. After that, the tubes were vortexed for 20 s, let to rest for 5 min, shaken 10–20 times and allowed to settle for 10 min. Then, 2 ml of the mixture was transferred into an Xpert MTB/RIF cartridge and tested for MTB according to the manufacturer's instructions. The leftover SR and transfer pipette tip were discarded to prevent cross-contamination. If the S1 sample’s result was unsuccessful, the remaining mixture was used to test again, or the testing procedure was repeated on the S2 sample.

In the reference laboratories, when the M samples arrived, laboratory technicians checked for adequate temperature and quality of the samples during transport. Each sputum collection tube was cleaned on the outside with 70% alcohol to remove any dust containing bacteria or fungi that could cause contamination during culture. The samples were decontaminated and inoculated in batches of maximum 14 samples. With each batch, a positive and negative control sample were taken along, in which the positive control was placed at the end and the negative control in the middle of the batch. Positive controls were saline spiked with a low MTB bacterial load (~ 50 to 100 bacteria/ml). For decontamination, the specimens were mixed with a double amount of 3% *N*-acetyl-l-cysteine-sodium hydroxide (NALC-NaOH), vortexed for less than 20 s, and after incubation for 15 min at room temperature, phosphate-buffered saline (PBS) was added up to 40 ml, and the mixture was centrifuged at 3000×*g* for 15 min. The supernatant of each sample was discarded, and the sediment was dissolved in 1 ml PBS, then the decontaminated specimen was inoculated (0.5 ml) into one liquid MGIT tube. The MGIT tube was gently shaken and allowed to settle for 30 min before being placed in the BACTEC MGIT 960 system. Incubated tubes were checked for growth for 42 days. If growth was detected, one drop of sediment was taken from the sample tube and stained using the Ziehl–Neelsen technique. Light microscopy was then used to detect cord factors and other acid-fast bacilli evidence in each Ziehl–Neelsen-stained sediment drop. MGIT growth was tested with the TBcID assay (Becton Dickinson, New Jersey, USA) to identify MTB. If there was no growth in a sample tube after 42 days of incubation, it would be classified as negative.

### Data collection and analysis

Data of Xpert were directly entered through laptops and tablets at each survey site using a locally developed, web-based data entry system. Culture results were recorded into logbooks at each laboratory, and then entered using Epidata Entry (The EpiData Association, Odense, Denmark). Data were analyzed using Stata14 (Stata Corporation, College Station, TX, USA). The primary outcome of this study was the comparison of overall positive/negative Xpert and MGIT results. Each Xpert positive test returns five Ct values of probe A–E, and the mean Ct value of each individual was calculated based on the mean of these five values. The bacterial load was reported as follows: very low (Ct value > 28), low (Ct value between 22 and 28), medium (Ct value between 16 and 22), and high (Ct value < 16) [19, 20]. When an Xpert-positive participant had two Xpert tests (S1 and S2) positive, we classified the results according to the highest bacterial load measured. To understand more about the characteristics of individuals with discordant test results, univariate and multivariate logistic regression were used to examine the association between discordant Xpert-MGIT results and sex, age, treatment history, chest X-ray result, TB symptoms, times to detection of MGIT, Xpert Ct value and reference laboratory. Discordances were compared in two ways: against both Xpert and MGIT positive (positive-positive analysis), and against both Xpert and MGIT negative (negative-negative analysis). Univariate regression was conducted first; multivariate models were constructed starting with all variables and excluding variables with p-values of higher than 0.2 in a step-wise manner. Numerical variables were checked for linearity using linear fitting, and independent variables in the model were tested for interaction. A full multivariate model with all potential factors was run as a sensitivity analysis.

## Results

**Fig. 1:**
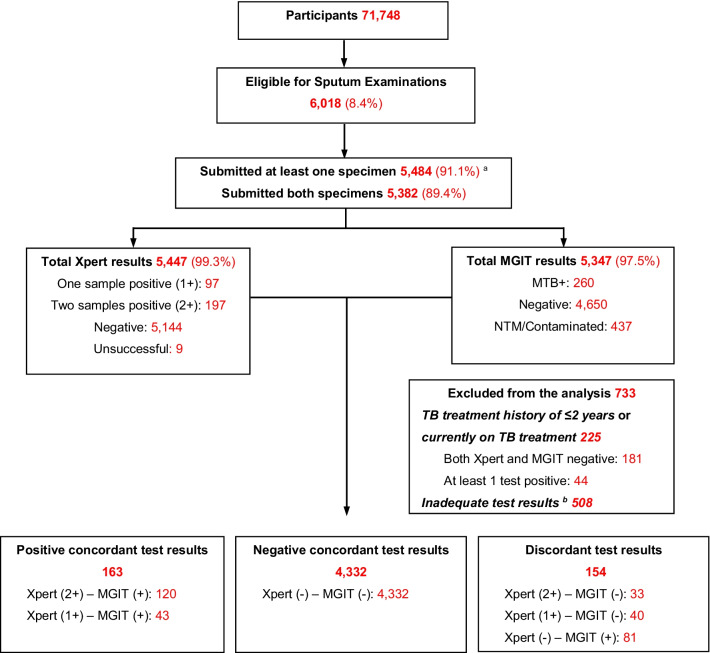
Summary of the laboratory result in the second national TB prevalence survey in Vietnam. *NTM* nontuberculous mycobacteria; *MGIT* Mycobacteria growth indicator tube; *Xpert* Xpert MTB/Rif. ^a^Submitted either the S (1 or 2) sample or the M sample, or both. ^b^Inadequate test results: missing either Xpert or MGIT result, or MGIT result was contaminated/nontuberculous mycobacteria

Of 71,748 participants screened for TB by oral interview and/or digital chest X-ray, 6018 (8.4%) screened positive and were eligible for sputum testing (Fig. 1). Of these, 5484 (91.1%) submitted at least one sputum sample (S or M) for TB testing, and 5382 (89.4%) provided both S1 and M samples. After excluding those currently on TB treatment or with a treatment history within 2 years (225 participants) and those whose test results were inadequate (508 participants), our study population was 4649 participants. At least one of two Xpert results was positive for 236 participants (5.1% out of 4649), in which 153 participants (3.3%) had positive results on both Xpert tests. MTB was detected in 244 morning sputum samples (5.3%) by MGIT culture. When comparing Xpert and MGIT results, 163 participants had concordant positive results, 4332 were negative on both tests, and 154 had discordant test results, which is Xpert positive—MGIT negative [(Xpert(+)MGIT(−)), or vice versa (Xpert(−)MGIT(+)—Fig. 1].

**Fig. 2:**
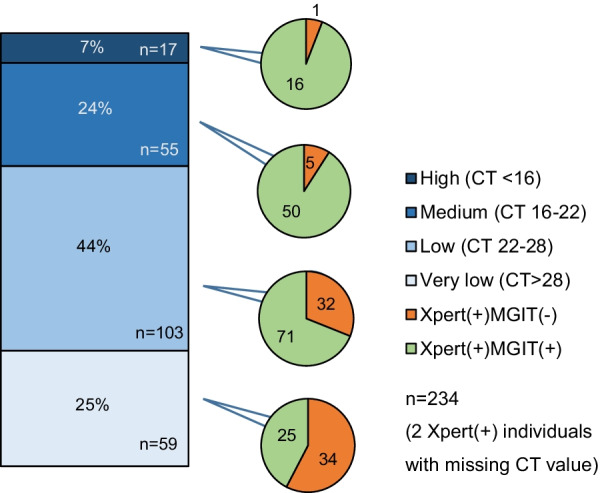
Bacterial load of Xpert-positive sputum samples, and their corresponding MGIT results. *CT* cycle threshold; *MGIT* Mycobacterium growth indicator tube; *Xpert*: Xpert MTB/Rif. n = 248 (2 Xpert(+) individuals with CT value missing)

**Fig. 3:**
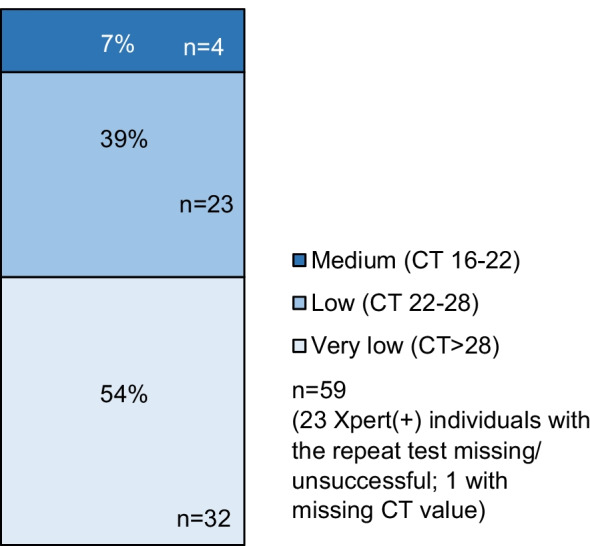
Bacterial load of Xpert-positive sputum samples for participants with discordance between their two Xpert tests. *CT* cycle threshold; n = 66 (15 Xpert(+) individuals with the repeat test missing/ unsuccessful; 1 with CT value missing)

Among Xpert(+) participants, Xpert(+)MGIT(−) results were more frequent when the bacterial load was lower, ranging from 1/17 (5.9%) in the highest to 34/59 (57.6%) in the lowest bacterial load category (p-value for trend < 0.001; Fig. 2). For those with positive Xpert results in their S1 samples and negative results in their S2 samples (n = 59, 18.6%), over half had a very low bacterial load (32 participants—54%), followed by those with a low bacterial load (23 participants—39%) and a medium bacterial load (4 participants—7%; Fig. 3).

Table 1 shows the multivariate association of participant characteristics and test results with discordant Xpert–MGIT results against concordantly positive Xpert-MGIT results (Xpert(+)MGIT(+)). Men were more likely to have Xpert(+)MGIT(−) than women (OR 2.8, p = 0.025). The odds for Xpert(+)MGIT(−) results was also higher among those who reported a history of TB treatment over 2 years ago (OR 2.7, p = 0.048) and among those reporting night sweats (OR 3.6, p = 0.024), and increased with the Ct value of Xpert (OR 1.2 per additional Ct value, p < 0.001). An Xpert(−)MGIT(+) result was associated with a longer time to detection of MGIT (OR 1.1 per additional detection day, p < 0.001). The number of participants in each category is presented in Additional file 1: Table S1.

**Table 1:** Multivariate association of Xpert-MGIT discordant sputum test results versus Xpert-MGIT concordant positive results with demographic and clinical variables and indicators of sputum bacterial load, among 317 Xpert or MGIT-positive participants of the second national tuberculosis prevalence survey in Vietnam

Factors	Xpert (+) – MGIT (−) (n = 73)		Xpert (−) – MGIT (+) (n = 81)	
	Adjusted OR (95% CI)	p-value	Adjusted OR (95% CI)	p-value
Male (vs. female)	**2.8 (1.1–6.8)**	**0.025**	0.7 (0.3–1.4)	0.284
Night sweats (vs. none)	**3.6 (1.2–11.3)**	**0.024**	0.5 (0.1–2.7)	0.416
Productive cough (vs. None)	0.4 (0.1–1.3)	0.113	0.3 (0.1–1.1)	0.316
Time to detect (day)	Not available		**1.1 (1.0–1.1)**	** < 0.001**
Xpert cycle threshold value	**1.2 (1.1–1.3)**	** < 0.001**	Not available	
Treatment history				
No treatment history	Ref		Ref	
TB treatment > 2 years	**2.7 (1.1–7.2)**	**0.048**	0.5 (0.2–1.6)	0.239

Odds ratios are adjusted for all other variables in the model. Bold numbers indicate statistical significance

Table 2 shows the multivariate association of participant characteristics and test results with discordant Xpert(+)MGIT(−) or Xpert(–)MGIT(+) results against concordantly MTB-negative Xpert and MGIT (Xpert(−)MGIT(−)) results. Again, the odds of Xpert(+)MGIT(−) was higher among men than women (OR 5.0, p < 0.001). A chest X-ray image suggesting active TB highly increased the odds of having one test positive (Xpert(+)MGIT(−) OR 67.9, p < 0.001; Xpert(−)MGIT(+) OR 13.8, p < 0.001). Those reported to have night sweats were also more likely to have Xpert(+)MGIT(−) compared with those with both tests negative (OR3.5, p = 0.002), while area (urban, remote, rural) and laboratory did not have a significant effect on the odds of having discordant test results. The number of participants in each category is presented in Additional file 1: Table S1.

**Table 2:** Multivariate association of Xpert- MGIT discordant sputum test results versus Xpert-MGIT concordant negative results with demographic, clinical and laboratory variables, among 4486 Xpert and MGIT-tested participants of the second national tuberculosis prevalence survey in Vietnam

Factors	Xpert (+) – MGIT (−) (n = 73)		Xpert (−) – MGIT (+) (n = 81)	
	Adjusted OR (95% CI)	p-value	Adjusted OR (95% CI)	p-value
Male (vs. female)	**5.0 (2.4–10.5)**	**< 0.001**	1.6 (0.9–2.6)	0.058
Night sweats (vs. none)	**3.5 (1.7–7.3)**	**0.002**	0.5 (0.1–2.3)	0.401
Abnormal chest X-ray (vs. none)	**67.9 (9.4–492.3)**	**< 0.001**	**13.8 (5.5–34.5)**	** < 0.001**
Area		0.114^a^		0.637^a^
Urban	Ref		Ref	
Remote	0.5 (0.2–1.1)	0.067	1.3 (0.7–2.3)	0.431
Rural	0.6 (0.4–1.1)	0.059	1.1 (0.6–1.8)	0.761
Laboratory		0.083^a^		0.798^a^
National referral lab	Ref		Ref	
Da Nang	1.4 (0.6–3.0)	0.450	0.7 (0.3–1.7)	0.490
Pham Ngoc Thach	1.0 (0.5–1.9)	0.998	1.2 (0.7–2.0)	0.614
Can Tho	1.8 (0.9–3.5)	0.062	1.0 (0.5–2.0)	0.903

Odds ratios are adjusted for all other variables in the model. Bold numbers indicate statistical significance

^a^Overall p-value of factors

## Discussion

In this community-based TB prevalence survey, we found that among all bacteriologically positive TB cases with adequate test results, nearly half (48.5%) had Xpert-MGIT discordant test results. For over one-quarter (28%) of Xpert-positive cases with both Xpert tests available, the repeat Xpert test remained negative. The majority of Xpert-positive results had low or very low bacterial loads (70%). Discordant test results were more frequent when the bacterial load was lower. Multivariate logistic regression against those with concordantly positive results showed a significant association between Xpert(+)MGIT(−) discordance and male sex, TB treatment history, lower Xpert bacterial load, and having night sweats, whereas Xpert(−)MGIT(+) discordance was associated with lower MGIT bacterial load only. Compared against concordantly negative results, factors associated with Xpert(+)MGIT(−) discordance were male sex, having night sweats, and abnormal chest X-ray result, whereas Xpert(−)MGIT(+) discordance was associated with abnormal chest X-ray.

The discordance rate of Xpert–MGIT results found in this study is higher than reported in studies done in clinical settings that included symptomatic patients who sought care in health centers or were diagnosed with TB in Brazil (20%) [13], South Africa (15%) [11] and South Korea (26%) [12]. In contrast, our study regarded active case finding in the general population. Indeed, 42.1% of the TB cases detected in our survey did not report any TB suggestive symptoms [18] and therefore would not qualify for sputum examination in routine health care. Symptomatic TB patients are more likely to expectorate sputum with high quality that on average contains more bacilli than asymptomatic patients [21]. The discordance rate found in our study is in line with other studies in community settings, for instance, in a TB prevalence survey in South Africa (75/212–35%), among a miners community in South Africa (97/214–45%) [22], or among prisoners in Ethiopia (12/22–55%) [23].

Two multivariate regressions were conducted to explore different aspects of the discordance between Xpert and MGIT results. The regression against Xpert(+)MGIT(+) can be interpreted as identifying the drivers of discordance, exploring which patient characteristic increase the odds of having discordant test results, while the regression against Xpert(−)MGIT(−) showed how likely the discordant test results identified a current TB patient. A driver of discordance in either way (Xpert(+)MGIT(−) or Xpert(−)MGIT(+)) was low bacterial load in the sputum specimen, suggesting that it reflects random variation between the two sputum specimens that each participant provided for Xpert and MGIT, whereby the bacterial load is just above the threshold of detection for either test. Also, for specimens with borderline bacterial load, any minor loss during transportation, storage or test processing could have resulted in Xpert(+)MGIT(−) discordance, as MTB growth in MGIT is vulnerable to loss of bacterial viability. Loss of viability will have less impact on the performance of Xpert, since traces of DNA are enough for Xpert to detect MTB, and the sample preparation process is simpler, which lowers the chance of losing bacteria [24]. The high proportion of paucibacillary specimens is thereby a likely explanation for the high discordance rate in our study. This is supported by our finding that discordance between the two Xpert test results mainly occurred if the positive one had a low or very low bacterial load. Among 154 participants with discordant test results, 117 (76.0%) were defined as TB cases by an expert panel using the chest X-ray result, symptoms and TB treatment history of each participant [18]. This highlights the role of bacterial load in the detection of MTB in the sputum samples, and that low bacterial load requires testing multiple samples, and multiple type of tests to detect TB.

Among individuals with positive Xpert results, the discordance rate was higher among males compared to females. Possibly, women comply better with instructions to expectorate sputum than men [25], producing higher quality sputum samples that contain enough live bacilli to grow in the MGIT system. Another driver of Xpert(+)MGIT(−) discordance was a history of previous TB treatment. This is in line with findings from other studies that non-viable bacterial DNA can persist in sputum samples of treated TB patients for up to 7 years after cure [26–28]. In these cases, it is uncertain to conclude that the Xpert results were false positive or these individuals were experiencing recurrent TB disease with low bacterial load, so they were referred to local district TB units for further diagnosis and treatment.

Having night sweats was associated with Xpert(+)MGIT(−) discordant results. As night sweats is not a specific symptom for TB, it may be that these were false-positive Xpert results in individuals who had other bacterial or viral infections [29]. However, this association could also reflect bias in our survey, as night sweats is not an objective symptom that can be easily distinguished from normal sweating, especially in a tropical climate like in Vietnam. Night sweats may also have interactions with unmeasured factors that explain this association. In any case, only a minority of participants with discordant test results had a history of TB or current night sweats, so their presence predicted only a limited proportion of the discordant results.

Against having Xpert(−)MGIT(−) results, determinants of having either a positive Xpert or a positive MGIT were male sex and abnormal chest X-ray. Both strongly suggest that either type of discordance truly reflected TB disease. The TB prevalence among men in our survey was four times higher than that among women [18]. Abnormal chest X-ray results suggestive of TB disease were also a screening criterion to determine who should get sputum tests done and have been reported to be strongly associated with pulmonary TB in many other studies [30, 31]. The numbers of participants with Xpert(+)MGIT(−) and Xpert(−)MGIT(+) are similar, suggesting that the sensitivity of one test is not clearly different compared to the other test.

Our study has several limitations. Firstly, the Xpert test was only repeated for those with positive initial test results (and for those with an initial unsuccessful test). Secondly, various factors might affect the positivity of both tests, including specimen quality, transportation, storage, sputum processing and weather conditions. Although most of Xpert instruments used in the survey were operated in an optimal environment and under optimal conditions, the tropical monsoon climate in Vietnam might have had an effect on the performance of Xpert during the survey, as the operating temperature of Xpert should be between 15 and 30 °C [32]. Finally, follow-up data was not available for many TB cases found in the survey, including those with discordant test results, which prevents the confirmation of TB status for such individuals.

## Conclusion

The findings of our study show a high rate of discordance in the performance of Xpert and MGIT to detect MTB in a community setting where the majority of identified TB patients had low bacterial loads. Rather than false-positive results, Xpert(+)MGIT(−) discordances in individuals without a history of previous TB treatment, especially among those with abnormal chest X-ray images, appear to primarily reflecting random between-sample variation around detection thresholds, and possibly loss of bacterial viability in the MGIT culture. In situations where the majority of TB cases are expected to have a low bacterial load, such as in community TB screening, childhood TB detection, or among patients with advanced immunocompromising diseases, discordant Xpert and MGIT results are more likely to occur and should be regarded true positive results. In these settings, multiple samples need to be tested and using only one diagnostic test may have too low sensitivity for detecting of MTB, even if that test is among the most sensitive diagnostic tests available. Also, culture laboratory quality assurance and adequate sputum transportation system are crucial for proper TB diagnosis.

## Supplementary Information

**Additional file 1: Table S1.** Number of participants with discordant and concordant test results, categorized by factors included in the multivariate analysis.

## Data Availability

The datasets used and analysed during the current study are available from the corresponding author (HVN) on reasonable request.
