# Supplementary material for: Discordant results of Xpert MTB/Rif assay and BACTEC MGIT 960 liquid culture to detect Mycobacterium tuberculosis in community screening in Vietnam
Source: BMC Infect Dis. 2022 May 31;22:506. doi: 10.1186/s12879-022-07481-5 (PMC9153144; doi:10.1186/s12879-022-07481-5)
Supplement: Supplementary file 1 — Additional file 1: Table S1. Number of participants with discordant and concordant test results, categorized by factors included in the multivariate analysis. [file 12879_2022_7481_MOESM1_ESM.pdf]

Table S1. Number of participants with discordant and concordant test results, categorized by factors included in the multivariate analysis.

Factors	Xpert(+)MGIT(-)<br>n (%)	Xpert(-)MGIT(+)<br>n (%)	Xpert(+)MGIT(+)<br>n (%)	Xpert(-)MGIT(-)<br>n (%)
**Sex**				
Male	65 (89.0%)	58 (71.6%)	123 (75.5%)	2613 (60.3%)
Female	8 (11.0%)	23 (28.4%)	40 (24.5%)	1719 (39.7%)
**Night sweats** [a]				
Yes	10 (13.7%)	2 (2.5%)	9 (5.5%)	268 (6.2%)
No	63 (86.3%)	79 (97.5%)	154 (94.5%)	4032 (93.8%)
**Productive cough** [a]				
Yes	38 (80.9%)	44 (86.3%)	111 (93.3%)	2951 (68.1%)
No	9 (19.1%)	7 (13.7%)	8 (6.7%)	1381 (31.9%)
**Chest X-ray** [a]				
Abnormal	72 (98.6%)	76 (93.8%)	160 (98.2%)	2239 (51.9%)
Normal	1 (1.4%)	5 (6.2%)	3 (1.8%)	2078 (48.1%)
**Treatment history**				
No treatment history	60 (82.2%)	76 (93.8%)	147 (90.2%)	3612 (83.4%)
TB treatment > 2 years	13 (17.8%)	5 (6.2%)	16 (9.8%)	720 (16.6%)
**Area**				
North	37 (50.7%)	28 (34.6%)	47 (28.8%)	1374 (31.7%)
Central	9 (12.3%)	19 (25.5%)	25 (15.3%)	1022 (23.6%)
South	27 (37.0%)	34 (42.0%)	91 (55.9%)	1936 (44.7%)
**Culture laboratory**				
National Referral Lab	19 (26.0%)	28 (34.6%)	51 (31.3%)	1820 (42.0%)
Da Nang	10 (13.7%)	7 (8.6%)	18 (11.0%)	443 (10.2%)
Pham Ngoc Thach	21 (28.8%)	31 (38.3%)	52 (31.9%)	1169 (27.0%)
Can Tho	23 (31.5%)	15 (18.5%)	42 (25.8%)	900 (20.8%)

[a] Participants who did not report symptoms or underwent chest X-ray examination were omitted.
